# The Difference in Achilles Tendon Loading within Immobilizing Boots Based on Ankle Angle, Boot Type, and Walking Speed

**DOI:** 10.1177/23259671241283806

**Published:** 2024-10-16

**Authors:** Todd J. Hullfish, Madison M. Woods, Michelle P. Kwon, Lorraine A.T. Boakye, Casey Jo Humbyrd, Josh R. Baxter

**Affiliations:** † Department of Orthopaedic Surgery, University of Pennsylvania, Philadelphia, Pennsylvania, USA; Investigation performed at the University of Pennsylvania School of Medicine, Philadelphia, Pennsylvania, USA

**Keywords:** ankle, Achilles tendon, foot, physical therapy/rehabilitation, biomechanics of tendon, gait analysis

## Abstract

**Background:**

Achilles tendon rupture is an increasingly common injury treated with progressive rehabilitation in an immobilizing boot. However, it is poorly understood how ankle angle, boot type, and walking speed affect Achilles tendon loading.

**Hypothesis:**

These different parameters would affect Achilles tendon loading in terms of (from greatest to least) ankle angle constraint, immobilization style, boot construction, and walking speed.

**Study Design:**

Descriptive laboratory study

**Methods:**

Ten healthy young adults (8 women and 2 men; age, 21 ± 2 years; body mass index, 21.5 ± 3.0 kg/m^2^) walked in 3 different immobilizing boots at self-selected slow, medium, and fast walking speeds. The authors estimated Achilles tendon loading using a 3-part instrumented insole within the immobilizing boot. The authors averaged tendon load across every stride for each condition and calculated 2-sided bootstrap confidence intervals. Peak tendon loading was compared across all boots, ankle angles, and walking speeds.

**Results:**

All boots and immobilization styles decreased tendon loading with respect to shod walking. Immobilization angle had the largest effect on tendon loading, followed by boot construction, and finally walking speed.

**Conclusion:**

Ankle angle, boot type, and walking speed can be modified to change loading progression during rehabilitation.

**Clinical Relevance:**

Understanding how immobilization affects tendon loading will enable clinicians to modify rehabilitation to improve functional outcomes.

Achilles tendon injuries are increasingly common in active adults and typically require restricted weightbearing while the tendon heals. For example, ruptures have increased 10-fold in the past 30 years.^13^ Recent advances in rehabilitative care have reduced rerupture rates to <5% regardless of whether patients are treated surgically or nonsurgically.^7,14^ However, nearly two-thirds of patients experience long-term functional deficits, and as many as 20% of patients are unable to return to physical activities they participated in before the injury.^2,21^ These functional deficits are explained by shorter plantarflexor muscle fascicles and an elongated tendon in patients,^12,15,16^ which are likely caused by suboptimal loading throughout tendon healing.^9,19^

Progressive rehabilitation with an immobilizing boot, as popularized by Willits et al,^20^ has been widely adapted for use in patients recovering from surgically or nonoperatively treated Achilles tendon ruptures, surgical debridement for chronic Achilles tendinopathy, and other Achilles tendon injuries. However, it is unclear how tendon loading varies between patients because of differences in immobilization protocols and physical therapy regimens. For Achilles tendon rupture, patients are treated either surgically or nonsurgically and then instructed to use a plantarflexed cast or splint for 2 weeks. They are then transitioned to an immobilizing boot with their ankles at 20° to 30° of plantarflexion for 8 to 12 weeks.^20^ Practitioners in the United States often prescribe immobilizing boots that were originally designed for the treatment of other foot and ankle injuries and later adapted for Achilles tendon rupture with the use of foam wedges or heel lifts. A newer boot style stabilizes the hindfoot with an articulating ankle joint constrained by a posterior strut instead of foam wedges. The variety of immobilization boots further complicates best practices for implementing progressive rehabilitation for patients recovering from Achilles tendon ruptures or debridement. Clinicians and patients also have access to a limited selection of immobilizing boots based on the contracted durable medical equipment vendor and insurance plans, which further complicates the standardization of immobilization protocols.

Estimating Achilles tendon loading during gait in an immobilizing boot is a technical challenge. Fröberg et al^6^ directly measured Achilles tendon loading using an invasive fiber optic technique, and Graf et al^8^ estimated net ankle moment in patients and controls while walking in different immobilizing boots. However, directly measuring tendon loading—as done by Fröberg et al—is impractical for clinical research and guiding rehabilitation. To address this clinical need, an instrumented boot paradigm was developed to quantify tendon loading within an immobilizing boot using a commercially available instrumented insole and a uniaxial load cell.^11^ This paradigm demonstrated that supporting the ankle in 30° of plantarflexion reduced Achilles tendon loading by 60% compared with walking in the boot with the ankle held at 0°. It is important to note that these findings were specific to an immobilizing boot with an articulating ankle joint constrained by a posterior strut. This posterior strut was the reason the boot could be instrumented with an additional sensor to validate the internal plantar pressure sensor paradigm. But patients are prescribed different types of immobilizing boots and given different rehabilitation instructions based on brace vendor availability and surgeon preference. It is unclear how these differing immobilizing boots and rehabilitation instructions—for example, guidance on changing the ankle angle or walking at different speeds—affect Achilles tendon loading.

The purpose of this study was to estimate Achilles tendon loading in 3 commonly prescribed immobilizing boots to understand how ankle angle, boot construction, immobilization style, and walking speed affected Achilles tendon loading. We tested 2 boot constructions, which we classified as hardshell and softshell, and 2 immobilization styles, which we classified as wedge and posterior strut immobilization. We also tested 3 self-selected walking speeds of slow, medium, and fast. We decided to perform this study in healthy controls to avoid any possible damage done to the healing tendon of patients when walking at faster speeds or with the foot in neutral position, which prior work shows generates loads in excess of 2.75 bodyweights.^10^ We hypothesized that these different parameters would affect Achilles tendon loading in terms of (from greatest to least) ankle angle constraint, immobilization style, boot construction, and walking speed. Establishing the effects of these clinically modifiable parameters on Achilles tendon loading is a necessary step toward developing precision rehabilitation strategies that optimize tendon healing and patient outcomes.

## Methods

### Study Design

Ten healthy young adults (8 women and 2 men; age, 21 ± 2 years; body mass index, 21.5 ± 3.0 kg/m^2^) participated in this institutional review board–approved study and provided written informed consent before participating. We recruited healthy adults with no history of Achilles tendon injury to establish the upper bounds of tendon loading under our test conditions without risking potential tendon damage. Participants walked across flat ground at self-selected slow, medium, and fast speeds in lab-standard running shoes (Air Pegasus; Nike) as well as 3 different immobilizing boots that were appropriately sized for their feet: Air Cam Walker (United Ortho), Air Select Walker (Aircast), and the VACOped (OPED Medical Inc). The Air Cam and Air Select boots use stacked foam wedges to position the ankle that are progressively removed during rehabilitation to decrease plantarflexion and gradually increase tendon load until the ankle is in neutral. The VACOped has an articulating ankle joint that is constrained by a plastic strut that can slide in a channel on the posterior of the boot. This boot constrains ankle motion via a pair of adjustable stops. Clinically, the bottom stop resists ankle dorsiflexion and is typically moved every 2 weeks to increase range of motion by 10°. We classified boots as having either a hard- or softshell construction and being a wedge or posterior strut immobilization style. We refer to the Air Cam Walker as the softshell wedge boot, the Air Select Walker as the hardshell wedge boot, and the VACOped as the hardshell posterior strut boot because our study focuses on the boot construction characteristics.

We measured plantar loading during each walking trial using an instrumented insole (Loadsol II acp; Novel) placed directly underneath the patient's foot to measure the normal forces within the shoe or boot. These data were logged at 100 Hz by streaming over Bluetooth to a handheld device (iPod Touch; Apple) using the insole's proprietary software. Patients were given time to acclimate to each boot before walking across flat ground until 15 to 20 strides per condition were collected. We then calculated Achilles tendon loading for each stride and resampled from heel-strike to toe-off. We resampled each stance phase to be 101 samples long using cubic spline interpolation. All patients performed the walking tasks in each boot, ankle angle, and walking speed condition in the same order to prevent data collection errors.

### Immobilizing Boots and Ankle Angles

**Figure 1.**
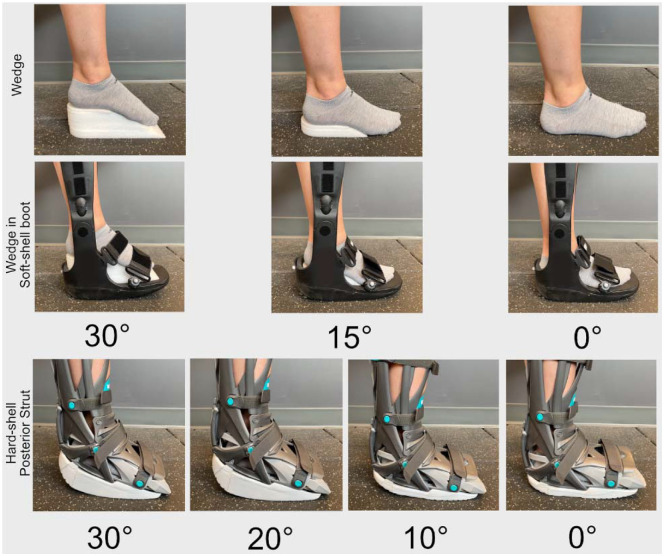
Participants walked in 3 different immobilizing boots, 2 of which are shown above. The softshell wedge (row 2) and hardshell wedge boots use stacked foam wedges to position the ankle. We used a 64-mm wedge for 30° of plantarflexion, a 23.5-mm wedge for 15° of plantarflexion, and no wedges for 0° of plantarflexion (row 1). The hardshell posterior strut boot (row 3) was positioned at 30°, 20°, 10°, and 0° of plantarflexion. The interchangeable cam bottom was swapped out for a flat bottom at 10°.

We compared peak tendon loading across 3 different immobilization conditions to understand how differences in boot construction and immobilization style affected tendon loading (Figure 1). We used a 64-mm wedge for 30° of plantarflexion, a 23.5-mm wedge for 15° of plantarflexion, and no wedges for 0° of plantarflexion for both wedge boots. These wedge conditions were based on the clinical timeline for ankle angle progression. We positioned the ankle at 30°, 20°, 10°, and 0° of plantarflexion for the posterior strut boot. We chose to make measurements at these ankle angles to characterize the full range for each style of boot. We found that ankle angle constraint correlated with Achilles tendon loading in preliminary testing, so we averaged the 20° and 10° posterior strut conditions to approximate 15° and directly compare tendon loading between the wedge and strut conditions.

### Achilles Tendon Loading

**Figure 2.**
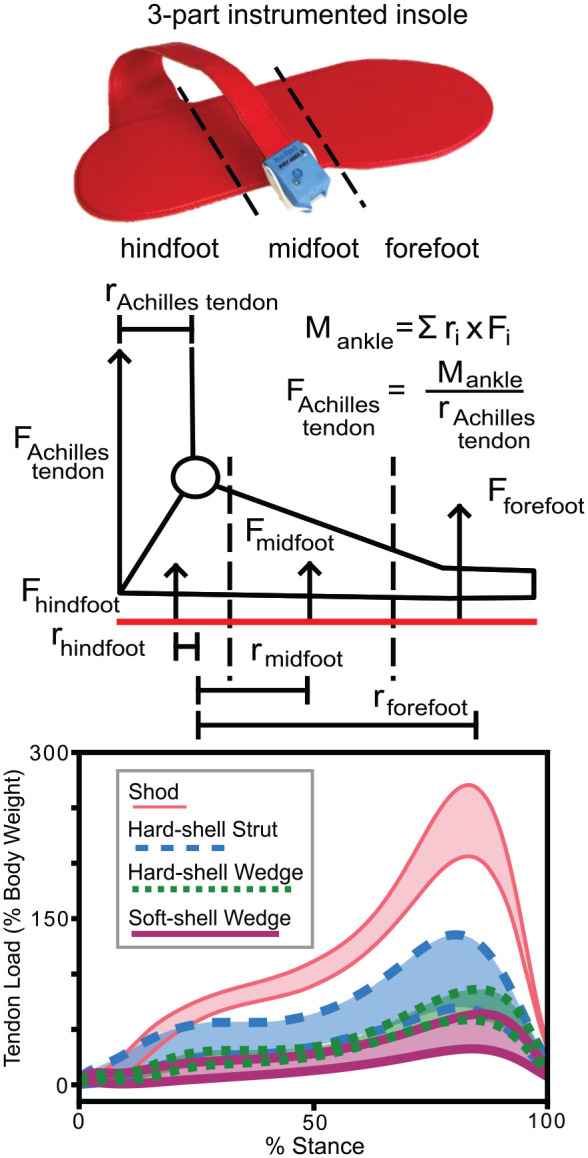
We estimated Achilles tendon loading using an instrumented insole algorithm. The insole (top) consisted of 3 force sensing zones under the hindfoot, midfoot, and forefoot. The moment about the ankle (middle) was quantified by summing the moments of each of these 3 zones together. Achilles tendon loading was then estimated by dividing this moment by the Achilles tendon moment arm. We then compared Achilles tendon loading during stance in the 3 immobilizing boots and in shoes (bottom).

We estimated Achilles tendon loading using a previously developed instrumented insole algorithm.^10,11^ The insole consists of 3 force-sensing zones under the hindfoot, midfoot, and forefoot (Figure 2). These zones are treated as discrete force transducers at constant distances from the ankle joint center. The ankle moment can then be quantified by summing the moments of each of these 3 zones together. Achilles tendon loading is estimated by dividing this moment by the Achilles tendon moment arm. This method is accurate to within 95% of gold standard inverse dynamic measurements in walking shoes.^10^ This method was similarly accurate in calculating Achilles tendon loading within an immobilizing boot.^11^ Using this instrumented insole algorithm, we calculated peak Achilles tendon loading during flat ground walking at self-selected slow, medium, and fast walking speeds in shoes and the 3 immobilizing boots at 3 clinically relevant ankle angles in the wedge boots and 4 clinically relevant ankle angles for the posterior strut boot. We then averaged these peak loads from every stride for each condition from each patient and calculated 2-sided bootstrap 95% CIs.

### Statistical Analysis

We compared peak tendon load in bodyweights across all boots, ankle angles, and walking speeds using 1-way, repeated-measures analysis of variance (ANOVA) and post hoc Tukey tests to determine how boot construction, immobilization style, and walking speed affect Achilles tendon loading. We tested our central hypothesis that loading would differ between boot construction and immobilization styles against the null at a significance level of α = .05. Boot construction, immobilization angle, and self-selected walking speed were all treated as categorical variables when comparing across test conditions. We reported *F* values, or the ratio of the explained variance to the unexplained variance in group means (larger *F* values indicate greater differences between groups), and associated *Pr > F* values for each ANOVA comparison as well as the post hoc *P* values calculated from the Tukey tests for each individual comparison group. We then calculated the percentage differences between each group using the general formula: (group B – group A)/group A, where group A is the group that we clinically expect to have lower tendon loading. For example, when progressing from 30° to 15° of ankle plantarflexion we calculated this percentage difference as (load at 15°– load at 30°)/load at 30°. We decided to instruct participants to walk at their self-selected slow, medium, and fast walking speeds to simulate how clinical instructions might be interpreted by a patient. We analyzed walking speed in 4 of the study participants by calculating the mean displacement of a heel marker during a stride divided by the stride time and found that participants walked at increasing speeds when given these instructions: slow (0.76-1.02 m/s), medium (1.2-1.5 m/s), and fast (1.6-1.7 m/s). We decided to test 10 healthy control participants to establish loading progression ranges for each boot, ankle position, and walking speed. All analyses were performed in Python 3.8 (Python Software Foundation) and ANOVA were performed using the statsmodels library (statsmodels; Version v0.14.4; statsmodels.org).

## Results

**Figure 3.**
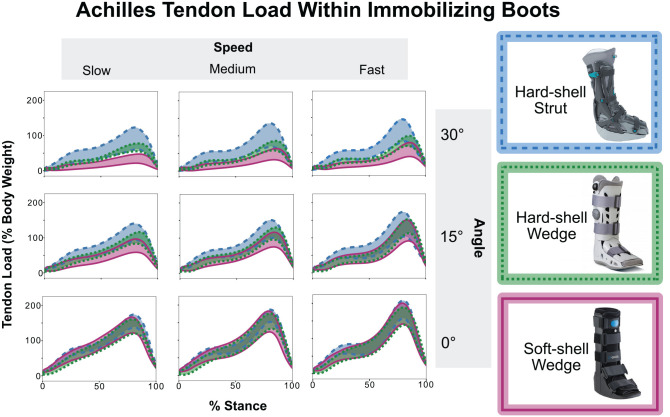
We calculated Achilles tendon loading during flat ground walking at self-selected slow, medium, and fast walking speeds in the 3 immobilizing boots at 3 clinically relevant ankle angles. Loading in these boots differed at 30° plantarflexion (top row). Tendon loading increased as we progressed the ankle angle from 30° to 0° and as we increased walking speed. At 0°, there were no statistically significant differences in loading between the boots (bottom row).

All 3 boots reduced Achilles tendon load significantly compared with the shod condition across the 3 ankle angles and speeds (*P* < .05). When comparing the 30° condition to shod walking at medium speed, the softshell wedge boot reduced tendon loading 79.5% (*P* < .001), the hardshell wedge boot reduced loading by 67.7% (*P* < .001), and the hardshell posterior strut boot reduced loading by 53.4% (*P* < .001). As we hypothesized, tendon loading increased as we progressed the ankle angle from 30° to 0° and increased walking speed from slow to fast. At 0°, tendon loading was comparable across all boots and walking speeds (Figure 3).

### Ankle Angle Within Boots

Tendon loading differed the most between ankle angles within the same boots (Table 1). The wedge boots were the most sensitive to changes in ankle angle across all walking speeds (*F* = 19.756-43.130; *Pr* > *F* < .001). On average, reducing plantarflexion within the boot caused tendon loading to increase by 48% in the hardshell wedge boot and 98% in the softshell wedge boot (Table 1). The hardshell strut boot was not as sensitive to changes in ankle angle, but the differences between all ankle angles were still significantly different (*F* = 4.852-7.282; *Pr* > *F* = .005-.021).

**Table 1:** Analysis of Variance Results Comparing Ankle Angle Within Each of the 3 Boots^*a*^

Softshell wedge	Post hoc *P* [% difference]		
	Slow	Medium	Fast
30°→ 15°	.056 [122.3]	.066 [101.2]	**.034 [110.7]**
15°→ 0°	**<.001 [104.5]**	**.003 [87.2]**	**.035 [62.1]**
30°→ 0°	**<.001 [302.6]**	**<.001 [231.9]**	**<.001 [198.4]**
*F* value	**43.130**	**23.220**	**19.756**
*Pr* > *F*	**<.001**	**<.001**	**<.001**
Hardshell wedge	Post hoc *P* [% difference]		
	Slow	Medium	Fast
30°→ 15°	**.016 [55.6]**	**.017 [57.7]**	**.011 [62.4]**
15°→ 0°	**.015 [45.9]**	**.012 [46.0]**	.119 [25.5]
30°→ 0°	**<.001 [119.6]**	**<.001 [124.9]**	**<.001 [107.5]**
*F* value	**41.278**	**32.034**	**23.157**
*Pr* > *F*	**<.001**	**<.001**	**<.001**
Hardshell strut	Post hoc *P* [% difference]		
	Slow	Medium	Fast
30°→ 15°	.463 [24.1]	.489 [21.8]	.473 [23.4]
15°→ 0°	.190 [31.6]	.157 [32.1]	.311 [25.5]
30°→ 0°	**.016 [66.6]**	**.013 [64.2]**	**.033 [57.4]**
*F* value	**7.120**	**7.282**	**4.852**
*Pr* > *F*	**.005**	**.005**	**.021**

a Significant values are denoted with bolded text. *P* values and calculated percentage differences are reported as *P* [% difference].

### Immobilization Style and Boot Construction

When comparing tendon loading between boots at the same ankle angles, we found that the only significant differences occurred at 30° and these differences decreased as ankle angle moved toward 0° (Table 2). Tendon loading at 30° was highest in the hardshell strut boot and lowest in the softshell wedge boot, differing by 42% on average across walking speeds (*P* = .007-.014). At 15°, tendon loading differed between the boots at slow (*F* = 6.960; *Pr > F* = .006) and medium walking speeds (*F* = 4.920; *Pr > F* = .020), but the post hoc tests showed the only significant difference was between the hardshell strut and softshell wedge boots at slow walking speed (*P* = .019). There were no differences between the boot constructions or immobilization styles when the ankle was at 0° (*P* > .05 for all conditions).

**Table 2:** Analysis of Variance Results Comparing 3 Different Boots at the Same Ankle Angle^*a*^

30°	Post hoc *P* [% difference]		
	Slow	Medium	Fast
Softshell wedge → hardshell wedge	.183 [86.6]	.366 [52.6]	.402 [42.3]
Softshell wedge → hardshell strut	**.007 [160.0]**	**.017 [113.3]**	**.014 [98.1]**
Hardshell wedge → hardshell strut	.289 [41.4]	.267 [43.3]	.213 [39.1]
*F* value	**7.814**	**7.636**	**10.375**
*Pr* > *F*	**.004**	**.004**	**.001**
15°	Post hoc *P* [% difference]		
	Slow	Medium	Fast
Softshell wedge → hardshell wedge	.293 [29.7]	.503 [19.3]	.857 [9.8]
Softshell wedge → hardshell strut	**.019 [56.5]**	.079 [38.5]	.429 [23.3]
Hardshell wedge → hardshell strut	.365 [20.7]	.505 [16.1]	.748 [12.3]
*F* value	**6.960**	**4.920**	2.144
*Pr* > *F*	**.006**	**.020**	.146
0°	Post hoc *P* [% difference]		
	Slow	Medium	Fast
Softshell wedge → hardshell wedge	.983 [4.4]	≥.999 [0.6]	.942 [–4.4]
Softshell wedge → hardshell strut	.964 [1.0]	.965 [1.3]	.999 [–0.1]
Hardshell wedge → hardshell strut	.902 [6.95]	.967 [2.4]	.923 [6.9]
*F* value	2.234	0.839	2.355
*Pr* > *F*	.142	.451	.129

a Significant values are denoted with bolded text. *P* values and calculated percentage differences are reported as *P* [% difference].

### Walking Speed

Walking speed also had a comparatively small effect on tendon loading (Table 3). At 30°, the softshell wedge boot was most sensitive to changes in walking speed (*F* = 18.932; *Pr > F* < .001), increasing tendon loading by 34% between slow and medium walking and 21% between medium and fast walking, but these increases in tendon loading were not significant. Walking speed had a similar effect on the hardshell wedge boot at 30° (*F* = 17.163; *Pr > F* < .001), but the increases in tendon load were lower in addition to being nonsignificant. The hardshell strut boot saw the smallest effect of walking speed across all ankle angles (*F* = 5.193-8.854; *Pr > F* = .002-.017) but had the most consistent changes in tendon loading. The lack of significant differences between any of the speed conditions in the post hoc tests while still seeing differences in the ANOVA results suggests that walking speed influences tendon loading; however, the variability between patients is quite high.

**Table 3:** Analysis of Variance Results Comparing Walking Speeds for Each of the 3 Boots and 3 Ankle Angles^*a*^

Softshell wedge	Post hoc *P* [% difference]		
	30°	15°	0°
Slow → medium	.591 [33.9]	.604 [21.7]	.832 [8.2]
Medium → fast	.685 [21.4]	.362 [25.6]	.650 [11.7]
Slow → fast	.182 [62.5]	.065 [52.9]	.320 [20.9]
*F* value	**18.932**	**6.916**	**9.634**
*Pr* > *F*	**<.001**	**.006**	**.001**
Hardshell wedge	Post hoc *P* [% difference]		
	30°	15°	0°
Slow → medium	.820 [8.6]	.650 [12.0]	.634 [11.3]
Medium → fast	.573 [13.4]	.404 [15.6]	.841 [6.2]
Slow → fast	.254 [23.1]	.090 [29.5]	.316 [18.2]
*F* value	**17.163**	**8.322**	**9.835**
*Pr* > *F*	**<.001**	**.003**	**.001**
Hardshell strut	Post hoc *P* [% difference]		
	30°	15°	0°
Slow → medium	.920 [10.1]	.902 [8.0]	.700 [8.0]
Medium → fast	.904 [10.1]	.780 [11.5]	.655 [8.1]
Slow → fast	.694 [21.2]	.517 [20.4]	.226 [16.8]
*F* value	**8.854**	**5.201**	**5.193**
*Pr* > *F*	**.002**	**.015**	**.017**

a Significant values are denoted with bolded text. *P* values and calculated percentage differences are reported as *P* [% difference].

## Discussion

In this study, we used an instrumented boot paradigm to estimate Achilles tendon loading across different immobilizing boot constructions, ankle constraints, and walking speeds. We found that Achilles tendon loading differed significantly between these boots when the ankle was placed in 30° plantarflexion and that all boots decreased load significantly when compared with shod walking. Loading differences between boots got smaller as ankle angle decreased and, at the 0° condition, these differences were not statistically significant. Increasing walking speed also increased tendon loading, but these differences were smaller than those associated with changing ankle angle or boot construction. Interestingly, the softshell wedge boot seemed to be more sensitive to changes in walking speed than the hardshell boots, but this is likely explained by the lower overall loading in the softshell boot.

This study has important clinical implications because it shows that boot selection will determine Achilles tendon loading early during healing. To our knowledge, most clinics in the United States follow some variation of a functional rehabilitation protocol popularized by Willits et al^20^ and recommended by clinical practice guidelines.^3^ These protocols are designed to reintroduce weightbearing early and help strengthen the healing Achilles tendon through controlled loading. The Willits protocol uses the hardshell wedge boot (AirCast) and specifically calls for a 2-cm heel lift to put the ankle in roughly 20° of plantarflexion. Other variations of this protocol, like the one tested in our study, instead use multiple 1-inch heel wedges to increase ankle angle to 30° of plantarflexion. Some protocols recommend specific amounts of load with which to increase weightbearing during rehabilitation. However, to our knowledge, there is no reliable way to measure these load changes without measuring plantar loading, which is typically not possible in the office setting. Our study demonstrated that boot selection materially affects tendon loading and varies in terms of which boot a clinician will prescribe; and how the clinician progresses the patient's ankle constraint and walking speed changes the loading the healing tendon undergoes. However, other clinicians across the globe may follow different protocols. Our study provides tendon loading estimations across a range of boot types, ankle angles, and walking speeds to provide clinicians with new evidence to better guide their practice.

**Figure 4.**
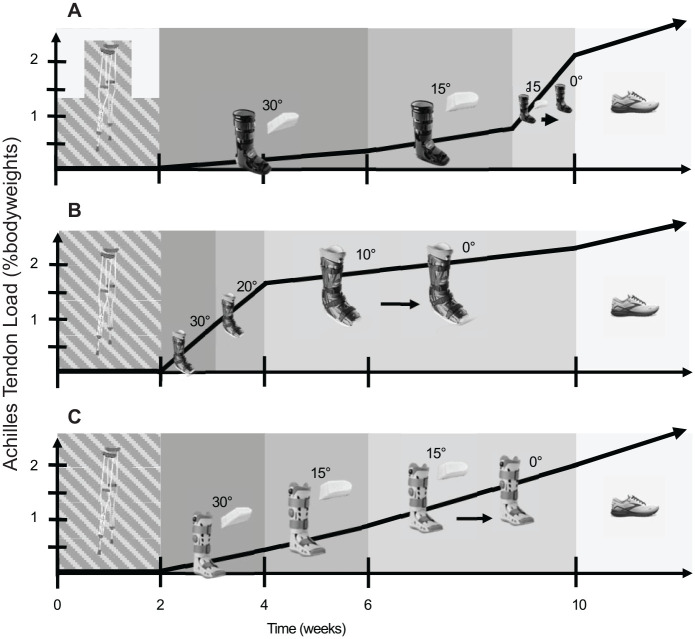
Estimating Achilles tendon loading during rehabilitation is a technical challenge. Using our results, we can project out what loading progressions might look like if the boots and immobilization styles were changed. The Willits protocol is the current clinical standard (c). This protocol could be made more conservative by swapping in the softshell wedge boot and keeping a patient at 30° for longer (a). In contrast, using the hardshell posterior strut boot and changing the ankle angle more quickly would result in a higher loading rehabilitation (b).

We feel it is important to understand how loading varies throughout Achilles tendon rupture recovery (Figure 4). The protocol laid out by Willits et al^20^ is moderate in terms of loading progression (Figure 4C), with patients continuing to use mobility aids such as crutches or canes throughout rehabilitation to help acclimate the healing tendon to loading throughout the first 4 weeks in the immobilizing boot. However, this loading progression could be made to progressively increase tendon loading less quickly by using a softshell wedge boot used in this study (Figure 4A) and delaying the progression of loading until later in rehabilitation. In contrast, a more aggressive loading profile could be achieved by using a hardshell posterior strut boot (Figure 4B). The higher early loading in this boot would theoretically stimulate the healing tendon^9^ and plantarflexors^19^ while preventing the excess dorsiflexion that is associated with rupture, making the eventual progression back into shoes less drastic. A randomized clinical trial by Aufwerber et al^1^ found that allowing immediate weightbearing in a posterior strut stabilized boot did not increase reruptures compared with delayed loading in a wedge boot. There may also be differences in optimal loading strategies between surgically and nonsurgically treated Achilles tendon ruptures, as nonsurgical treatment might necessitate keeping the ankle in greater amounts of plantarflexion for longer periods of time than surgical treatment. Our study does not identify which loading strategy is best but makes 2 important contributions. First, we provided quantitative guidelines to develop tendon loading progressions based on clinician experience and patient factors. Second, we established Achilles tendon loading ranges across different boot constructions, ankle constraints, and walking speeds. We expect that these findings will support future clinical trials aimed at optimizing tendon healing by establishing clinically modifiable instructions that reliably proscribe tendon loading.

Our study suggests that immobilization style is an important driver of tendon loading throughout healing. While the wedges we used in this study are not universal, the style of wedge is comparable with those frequently used in the United States. These wedges constrain ankle angle by prescribing the distance between the heel and the bottom of the shoe (Figure 1). Compared with an articulating ankle-foot orthosis such as the posterior strut boot (VACOped), which prescribes the position and range of motion of the boot ankle joint, this style of wedge seems to modify the angle between the hind- and midfoot rather than the angle of the ankle. As a result, this seems to protect the healing Achilles tendon by driving loading through the heel rather than through the toe and reducing tendon force. This difference in ankle constraint is highlighted in a study performed by Ellison et al,^5^ where they quantified ankle angle within several different boots using lateral radiographs. Additionally, findings from Zellers et al^22^ show that increasing wedge height reduces muscle activity in the plantarflexors. These findings increase our confidence that the reductions in tendon loading we see in the wedge boots are caused by driving loading through the heel.

Previous studies have sought to quantify the differences in Achilles tendon loading in an immobilizing boot using different techniques. One study, performed by Fröberg et al,^6^ used an invasive fiber optic technique to make measurements of Achilles tendon load in an immobilizing boot that was modified to expose the tendon. They found that tendon force increased compared with barefoot walking while the ankle was held in 20° of plantarflexion by an articulating ankle-foot orthosis-style boot. This finding is opposite of our current results, which found all boots reduced loading compared with shod walking. This is likely explained by the differences in boot construction, as the boot used in the Fröberg et al study had a flat sole that was not adjusted when the foot was in plantarflexion, forcing loading through the forefoot of the boot and increasing ankle moment. In contrast, the posterior strut boot used in our study has an interchangeable cam bottom that helps the foot roll during stance when the ankle is placed in ≥20° of plantarflexion. This cam bottom promotes a more natural gait pattern,^8^ with the center of pressure moving from hind- to forefoot throughout stance.^17^ Another study, performed by Sommer et al,^17^ characterized gait parameters and boot-ankle net moment for 3 different boots, including the hardshell posterior strut boot used in this study. Sommer et al found that each of the boots they tested reduced net ankle moments compared with barefoot walking. It is important to note that net ankle moment is the summation of the loads carried by the biologic tissues—in this case, the Achilles tendon—and the loads carried by the immobilizing boot.^11^ Sommer et al also quantified the range of motion of the ankle within the immobilizing boots and showed that the hardshell posterior strut boot constrained the most ankle motion, suggesting that the posterior strut design increases boot rigidity.

We decided to study the effects of self-selected walking speed rather than having patients walk at a set speed. This was primarily to match how a clinician would use walking speed to modify tendon loading, as we are currently not aware of any way to reliably prescribe set walking speeds in the real world for patients recovering from Achilles tendon rupture. Our results suggest that increasing walking speed does in fact increase tendon loading on average, but these changes are highly variable between patients. Previous work has demonstrated that increasing walking speed does increase tendon loading,^4^ but the increases due to walking speed we found were relatively small in comparison with the increase in tendon load with decrease in plantarflexion within a boot. Further work into the effects of walking speed on tendon loading may be useful for optimizing rehabilitation in an immobilizing boot, but our results suggest that changing other parameters such as ankle angle progression and immobilization style will show less variability between patients.

### Limitations

There are several important limitations to consider when interpreting our results. First, we measured tendon loading in healthy controls as opposed to in patients with tendon ruptures. We decided to study noninjured participants for 2 reasons: (1) to compare boot type, ankle angle, and walking speeds within individuals without putting injured patients at risk of excessive loading and (2) to establish loading upper bounds for each test condition. We considered this an important aspect of our study because patients are likely to walk with some guarding, dependent on their fear of movement and healing status. Second, 80% of our patients were women even though women only account for roughly 15% of patients with Achilles tendon rupture.^18^ However, we did not find any evidence in the literature suggesting that male and female patients load their healing tendons differently throughout rehabilitation. Additionally, our patients were younger and had lower body mass indexes than the typical patient with Achilles tendon rupture, and these differences may affect walking speed and tendon loading. For instance, it is possible in extreme cases that patients with very high body mass indexes could have large amounts of soft tissue on their shanks that could cause fit issues with the boot. However, because we are measuring changes in tendon loading within individual patients, we find it unlikely that these factors would lead to different Achilles tendon loading across the mechanical constraints that are introduced by the boots we tested. We did not increase our statistical power to detect subtle differences in peak tendon loading that result from the complex interaction between these parameters because we do not currently have the necessary evidence to state that one loading progression is better than another. Our future work is aimed at establishing “loading profiles” that lead to optimal structural, functional, and patient-reported outcomes. The transition from a neutrally aligned boot to a walking shoe is important, but we did not include a shoe with heel lift condition because the added time was impractical. Last, we developed our instrumented boot paradigm using an instrumented version of the hardshell posterior strut boot but were unable to implement a similar instrumentation scheme in the other 2 boots because of the nature of their construction. As a result, it is possible that we were underapproximating tendon loading within the wedge boots; but this is unlikely, as the neutral conditions for each boot had very similar loading levels where ankle constraints were most similar.

## Conclusion

Improving rehabilitation after Achilles tendon injuries is a critical step toward improving long-term functional outcomes for patients. While we do not fully understand what levels of loading are optimal for healing individual patients, current rehabilitation guidelines adequately protect patients from rerupture. Our results demonstrated that loading within immobilizing boots can be modified by the treating clinicians and rehabilitation team to fit the needs of individual patients and their unique injury scenarios.

Our study showed that ankle constraint delivered by boot construction and immobilization style along with walking speed govern Achilles tendon loading. These factors were clinically modifiable by the treating clinician—either by changing the types of immobilizing boots prescribed to patients or by more specific instructions to the patients on how to ambulate in these boots.
